# Forbidden Conversations: A Comprehensive Exploration of Taboos in Sexual and Reproductive Health

**DOI:** 10.7759/cureus.66723

**Published:** 2024-08-12

**Authors:** Nor Faiza Mohd. Tohit, Mainul Haque

**Affiliations:** 1 Department of Community Health, Universiti Pertahanan Nasional Malaysia (National Defence University of Malaysia), Kuala Lumpur, MYS; 2 Department of Research, Karnavati Scientific Research Center (KSRC) School of Dentistry, Karnavati University, Gandhinagar, IND; 3 Department of Pharmacology and Therapeutics, Universiti Pertahanan Nasional Malaysia (National Defence University of Malaysia), Kuala Lumpur, MYS

**Keywords:** policy recommendations, human rights, healthcare access, sexual orientation, contraception, menstruation, cultural beliefs, stigma, reproductive health, sexual health

## Abstract

This scoping review aims to comprehensively explore the landscape of taboos and their impact on sexual and reproductive health. Titled “Forbidden Conversations,” it delves into the intricate web of societal, cultural, and religious norms that have contributed to the elusive and often stigmatized nature of sexual and reproductive health topics. The review navigates through the multifaceted dimensions of these taboos, shedding light on their impact on individuals, communities, and public health while advocating for a paradigm shift toward open, inclusive, and informed dialogue. The analysis within this review spans a decade, capturing the most recent and relevant literature to map the landscape of taboos in sexual and reproductive health. It explores the persistent societal apprehensions and subsequent stigmatization surrounding topics such as menstruation, contraception, fertility, sexual orientation, and gender identity. The review contextualizes the multifaceted challenges presented by these prevailing norms by tracing historically rooted taboos and their evolution across different cultures and contexts.

The scoping review identifies the profound implications of these taboos on public health, highlighting how they contribute to disparities in access to healthcare, perpetuate misinformation, and infringe upon the fundamental rights of individuals. It addresses the challenges in sexual education, emphasizing how these taboos impede comprehensive understanding and enforcement of sexual and reproductive health rights among adolescents and young adults. The intersectional approach taken in this review situates these taboos within broader systems of inequality, emphasizing the compounded impact they have on marginalized populations. Through this comprehensive exploration, the review aims to provide actionable insights and identify existing research, policy, and practice gaps. It seeks to lay the foundation for future initiatives that advocate for destigmatization, empowerment, and equity in sexual and reproductive health. Ultimately, “Forbidden Conversations” aims to steer the conversation toward openness and inclusivity while advocating for unbiased, comprehensive sexual and reproductive healthcare with dignity for all individuals.

## Introduction and background

In the intricate tapestry of human societies, taboos surrounding sexual and reproductive health (SRH) remain powerful forces that shape cultural norms, influence behavior, and impact health outcomes [1]. Despite progress in global health education, the topics of sex and reproduction continue to be shrouded in secrecy, misinformation, and stigma [2]. This review seeks to unravel these complexities, fostering a broader understanding and encouraging open dialogue on subjects often relegated to the shadows of discourse. SRH is a fundamental aspect of human well-being, encompassing not only the absence of disease and infirmity but also the presence of positive and respectful relationships, safe and fulfilling sexual experiences, and the ability to freely make choices about one’s reproductive life (World Health Organization (WHO), 2017) [3]. However, societal taboos often obstruct access to accurate information and essential services, perpetuating ignorance, fear, and discrimination. These taboos vary widely between cultures and contexts, profoundly affecting individuals’ health, rights, and quality of life.

Data from various sources highlight the tangible impact of SRH taboos on public health outcomes. For example, An estimated 16 million adolescents aged 15-19 give birth yearly. Complications from pregnancy and childbirth are the leading cause of death in girls aged 15-19 in low and middle-income countries (LMICs), where almost all of the estimated 3 million unsafe abortions occur [4]. In many Sub-Saharan African countries, the stigma around human immunodeficiency virus (HIV)/acquired immunodeficiency syndrome (AIDS) continues to hinder testing and treatment uptake. According to the Joint United Nations Programme on HIV/AIDS (UNAIDS), only 81% of people living with HIV knew their status in 2020, highlighting the gap caused by stigma and fear of discrimination (UNAIDS, 2020) [5]. In the United States, a study by Mark and Wu (2022) found that funding for more comprehensive sex education led to an overall reduction in the teen birth rate at the county level of more than 3% [6]. This underscores the role of open dialogue and education in improving SRH outcomes. Historically, discussions on topics such as contraception, menstruation, sexually transmitted infections (STIs), and sexual orientation have faced considerable censorship and societal disdain. This reluctance has persisted despite international efforts to promote comprehensive sexual education and reproductive health services (WHO, 2019) [7]. For instance, in many parts of the world, menstruation is still considered a topic not to be openly discussed, leading to “period poverty” and a lack of menstrual hygiene management [8].

Period poverty refers to the lack of access to menstrual products, education, washing facilities, and waste management. It embodies the economic, social, and cultural obstacles that hinder individuals from managing their menstruation safely, comfortably, and with dignity. This phenomenon is deeply intertwined with issues of gender inequality, education, health, and economic disparity, and it affects millions of menstruators worldwide [9,10]. Period poverty is exacerbated by a pervasive lack of SRH education. In many cultures, menstruation is a subject of significant taboo, leading to the widespread dissemination of myths and misconceptions [11]. These taboos often prevent meaningful dialogue and education about menstrual health, leaving young people unprepared to manage their menstruation effectively [12]. For instance, according to the WHO, comprehensive sexuality education, which includes menstruation, remains limited in many contexts, contributing to misinformation and stigma [7]. The absence of SRH education extends beyond the mere mechanics of menstruation to broader issues of reproductive and sexual health. This lack of knowledge can have cascading effects, contributing to early pregnancies, unsafe abortions, and an increased risk of STIs (UNESCO, 2018) [13]. Addressing period poverty, therefore, requires integrating comprehensive SRH education into school curricula and public health initiatives [9,11]. The stigma surrounding STIs can deter individuals from seeking diagnosis and treatment, exacerbating public health issues (WHO, 2016) [14]. Moreover, the taboo surrounding contraception and family planning often impedes women’s autonomy over their reproductive choices, leading to unplanned pregnancies and unsafe abortions. The WHO (2020) reports that about 214 million women of reproductive age in developing regions who want to avoid pregnancy are not using a modern contraceptive method, a situation rooted in both lack of access and prevailing cultural and religious norms [15].

**Figure 1:**
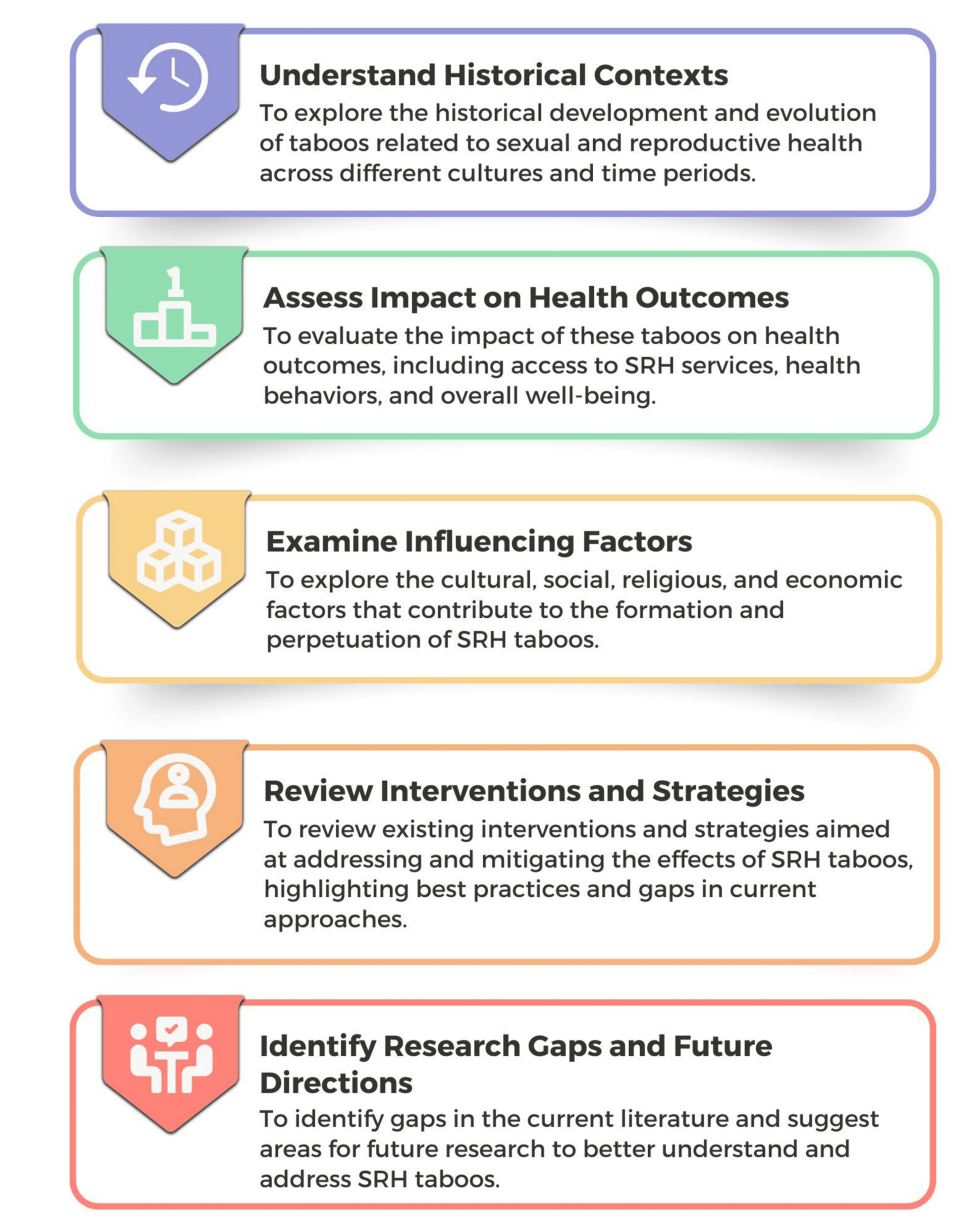
Objectives of the scoping review. Image credit: Nor Faiza Mohd. Tohit.

The objectives of this review are multifaceted (Figure 1). First, it aims to comprehensively understand the cultural, societal, and economic factors that underpin taboos in SRH. This involves exploring regional and cultural variations in these taboos, shedding light on the diverse challenges different populations face, and highlighting the societal influences that drive these variations. Additionally, the review addresses critical knowledge gaps and misconceptions, providing a more accurate understanding of these crucial topics while dispelling myths and misinformation. It also aims to emphasize the intersectionality of SRH with gender, sexuality, and socioeconomic status, acknowledging the compounding effects of multiple forms of discrimination and inequity.

## Review

Methodology

**Figure 2:**
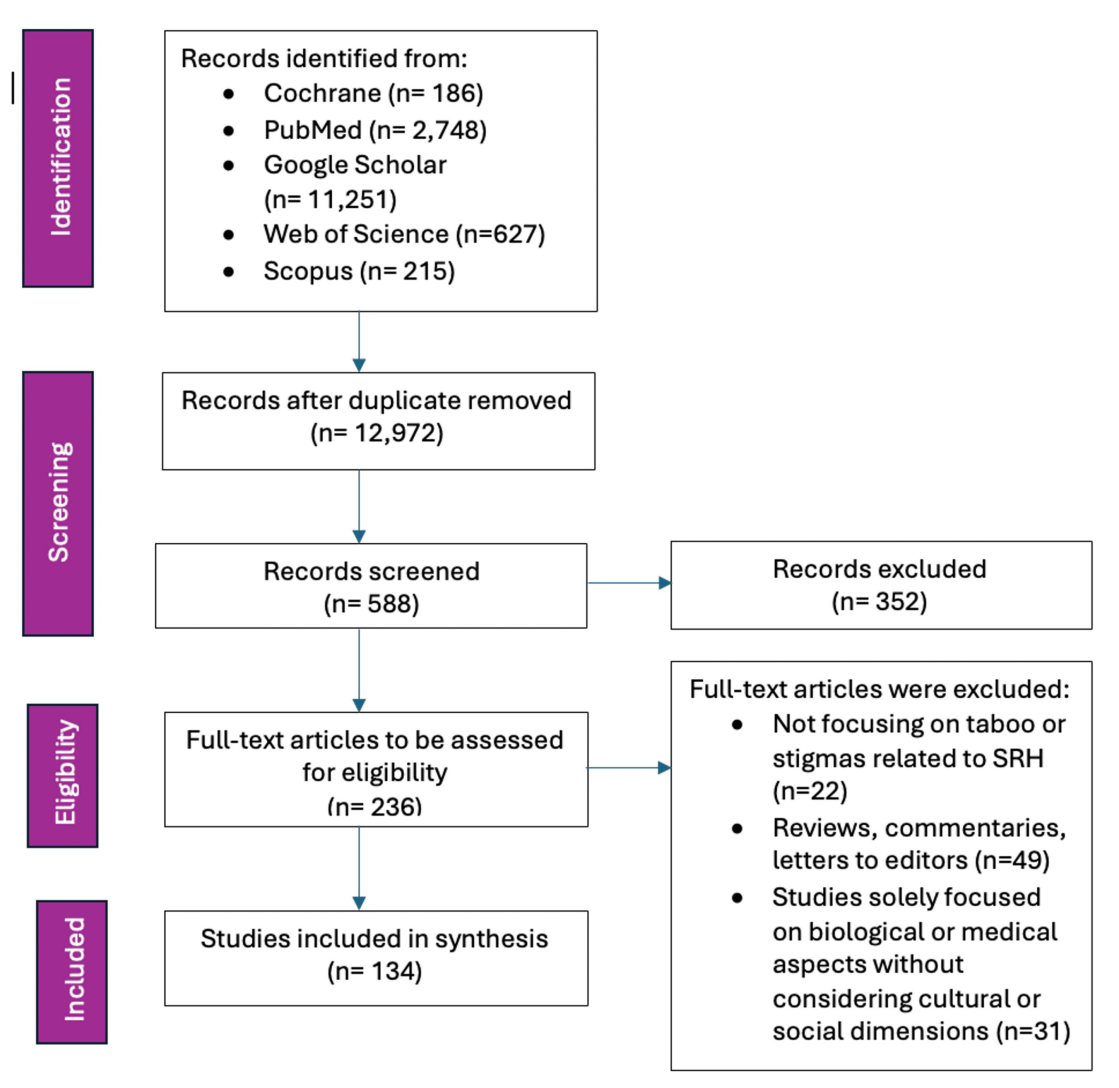
Preferred Reporting Items for Systematic Reviews and Meta-Analyses Extension for Scoping Reviews (PRISMA-ScR) flow diagram. Image credit: Nor Faiza Mohd. Tohit.

This scoping review adopted a systematic approach, following the framework outlined by Arksey and O’Malley (2005) (Figure 2) [16]. Additionally, the methodology adhered to the Preferred Reporting Items for Systematic Reviews and Meta-Analyses Extension for Scoping Reviews (PRISMA-ScR) guidelines [17] to ensure transparency and rigor in the reporting process.

A comprehensive search was conducted across multiple academic databases, including PubMed, Scopus, Web of Science, and relevant social science and public health repositories. The search incorporated Medical Subject Headings (MeSH) and other applicable terms to capture a wide array of literature related to taboos in SRH. Key MeSH terms and search keywords included “Sexual Health,” AND “Reproductive Health,” AND “Taboos,” AND “Stigma,” AND “Menstruation,” AND “Contraception,” AND “Cultural Beliefs,” AND “Sexually Transmitted Infections,” AND “Family Planning Services.”

The review included peer-reviewed journal articles, grey literature, policy documents, and reports from international health organizations such as the WHO. Studies focusing on taboos and stigmas in SRH, including qualitative or quantitative research, reviews, or theoretical papers, and published in English were selected. No restrictions were placed on the publication date to encompass historical and contemporary perspectives. Articles that did not directly address taboos or stigmas related to SRH, opinion pieces without empirical or theoretical grounding, and studies solely focused on biological or medical aspects without considering cultural or social dimensions were excluded. A standardized form was utilized to extract essential information from selected studies, including study objectives, methods, population, context, findings, and cultural aspects. The extracted data were mapped and categorized to identify common themes and patterns. In line with the Arksey and O’Malley framework, a narrative synthesis approach was employed to present these themes, ensuring a comprehensive overview of identified taboos and their impacts.

Multiple reviewers independently screened titles, abstracts, and full texts to determine eligibility, ensuring consistency and minimizing bias. Discrepancies were resolved through discussion or consultation with a third reviewer. The quality and potential biases of the included studies were appraised using established assessment tools. By following the PRISMA-ScR guidelines, the review process remained transparent and replicable, enhancing the reliability and validity of the findings. Through this meticulous and inclusive methodology, the scoping review provided a nuanced understanding of the cultural, societal, and economic influences shaping taboos in SRH. The insights gained informed public health initiatives, community interventions, and academic research, promoting more inclusive and informed discussions, policies, and practices that uphold the rights and well-being of all individuals in SRH.

Literature review

Historical Context: How Taboos in Sexual and Reproductive Health Have Evolved Over Time

The evolution of taboos in SRH is deeply embedded in the cultural, religious, and societal fabric of human history. These taboos, often reflecting prevailing moralities and power structures, have profoundly influenced the management and discourse surrounding SRH throughout the ages.

Mysticism and control in ancient civilizations: SRH was frequently shrouded in mysticism and strict moral codes. For instance, in ancient Mesopotamia and Egypt, menstruation was often seen as a powerful and somewhat perilous event, leading to the isolation of menstruating women due to perceived impurity [18]. The control of women’s reproductive capabilities was also prevalent, with early forms of contraception often linked to ritualistic practices.

Philosophical and medical insights during classical antiquity: Greek and Roman societies exhibited a complex mix of perspectives on SRH during classical antiquity. Notable thinkers such as Hippocrates and Galen began to move toward a more systematic and medical understanding of these issues. However, societal norms still enforced strict boundaries. Patriarchal structures predominantly controlled women’s sexuality, and discussions of sexual health were largely confined to male-dominated scientific discourse [19].

Religious dominance and suppression in the Medieval era: Europe saw the entrenchment of religious dogma, particularly by the Christian Church, which began to exert considerable influence over sexual and reproductive norms in the Medieval era. Celibacy and virginity were highly esteemed, and sexual activity was essentially sanctioned only within the bounds of marriage for procreation purposes [20]. Menstruation, childbirth, and sexual practices were often viewed through a lens of sin and purity, further reinforcing silence and shame around these topics [21].

Questioning norms in the time of Renaissance and Enlightenment: A slow, nonetheless considerable, shift began during the Renaissance and Enlightenment eras. Humanism and the questioning of religious authority permitted a more open exploration of human sexuality and reproduction. Figures such as Andreas Vesalius and William Harvey made strides in understanding human anatomy and reproduction [22]. Despite advancements, many taboos persisted, particularly those surrounding female sexuality and reproductive rights.

The rise of medicalization in the 19th century: Intense medicalization of SRH was introduced in the 19th century. This era saw the emergence of obstetrics and gynecology as distinct medical fields, along with a heightened interest in controlling reproductive behaviors through science. Practices such as forced sterilizations and the stigmatization of non-procreative sexual behaviors flourished, influenced by eugenics and social purity movements [23]. Although medical knowledge expanded, it often reinforced existing taboos and gender biases.

Waves of change in the 20th century: In the early 20th century, human beings witnessed substantial efforts to challenge and transform SRH taboos. The women’s rights movement, the advent of birth control, and the sexual revolution of the 1960s played pivotal roles. Activists such as Margaret Sanger advocated for contraception, leading to greater autonomy for women over their reproductive health [24]. The second half of the century saw the destigmatization of topics such as menstruation and homosexuality, although resistance from conservative sectors persisted.

Continuing evolution throughout the 21st century: Discussions surrounding SRH have become increasingly inclusive and destigmatized, although considerable challenges remain in the 21st century. Global health organizations such as the WHO have critically advocated for comprehensive sexual health education, reproductive rights, and gender equality [4]. Online platforms have facilitated more open dialogues, challenging previously entrenched taboos. However, the backlash from conservative and religious groups continues to influence policy and social attitudes in many regions. The evolution of taboos in SRH vividly illustrates a complex interplay between cultural, religious, and scientific influences over the centuries. While significant progress has been made in destigmatizing these critical areas of human health, ongoing efforts are required to challenge further and dismantle enduring taboos. By understanding the historical context, we can better appreciate the strides made and recognize the work still needed to achieve a more open and equitable discourse on SRH.

Cultural and Societal Perspectives on Taboo Topics in Sexual and Reproductive Health

SRH is an essential component of overall well-being, yet discussions often remain concealed under layers of cultural and societal taboos [25,26]. These taboos are deeply rooted in historical, religious, and sociocultural contexts, varying significantly across different societies [27,28]. They influence personal beliefs, behaviors, and broader public health policies and educational programs [29].

Historical and religious foundations: Many cultures have historically ascribed moral and spiritual meanings to sexual and reproductive functions. In several ancient civilizations, menstruation was considered both powerful and polluting, leading to the isolation of menstruating women [30]. These notions of purity and impurity persist in various forms today. In many Hindu communities, menstruating women are still restricted from participating in certain religious activities and entering temples during their period [31]. Religions have played a substantial role in shaping sexual mores. Christianity, for example, traditionally promoted celibacy and sex only within marriage, primarily for procreation, viewing other sexual acts as sinful [32]. These teachings have had lasting impacts on Western societies, contributing to the stigmatization of premarital sex, homosexuality, and contraceptive use. Similarly, in Islam, while sex within marriage is encouraged, discussions around sexuality and reproductive health often remain private, with significant taboos surrounding contraceptive use and premarital sex [33].

Cultural norms and gender roles: Perceptions of SRH are appreciably influenced by cultural norms and gender roles. Societies with rigid gender norms often exert control over women’s sexuality and reproductive choices, reflecting broader power dynamics. In many cultures, notions of honor and virginity are profoundly tied to women’s behavior, leading to practices such as chastity belts in Medieval Europe and honor killings in some modern-day societies [34]. These cultural perspectives can also affect men’s reproductive health. In many societies, masculinity is often associated with sexual prowess and fertility, leading to the stigmatization of issues such as infertility and STIs among men [35]. Such stigmas can prevent men from seeking necessary health services, further entrenching taboos.

Educational and policy implications: The cultural and societal taboos surrounding SRH inevitably influence educational content and public health policies. In many countries, sex education in schools is limited or non-existent due to cultural sensitivities and political resistance. The United States, for instance, has seen significant debate over abstinence-only versus comprehensive sex education, with the former often rooted in cultural and religious values [36]. In contrast, countries with more open attitudes toward sexual health, such as the Netherlands, which emphasizes comprehensive sex education, report lower rates of teen pregnancies and STIs [37]. This illustrates how cultural contexts are shaped by policy decisions, reflecting varying degrees of comfort and openness regarding sexual and reproductive topics.

Modern movements and shifting perspectives: The taboo topics regarding SRH have begun to be challenged through modern movements and shifted perspectives. The feminist movement has been pivotal in advocating for women’s reproductive rights, including access to contraception and safe abortion services [38]. Global health organizations such as the WHO promote a rights-based approach to SRH, emphasizing the importance of education, access to services, and the elimination of stigma and discrimination [3]. Social media and the internet have also provided platforms for more open and inclusive conversations, enabling individuals to share experiences and access information previously obscured by taboos [39]. Cultural and societal perspectives on taboo topics in SRH are deeply entrenched and multifaceted, influenced by historical, religious, and sociocultural factors. Significant challenges remain while progress has been made in destigmatizing and bringing these issues into public discourse. Understanding these perspectives is crucial for developing effective policies, educational programs, and public health initiatives that respect cultural sensitivities while promoting the health and rights of all individuals.

Impact of Taboos on Individuals and Communities

Tackling taboos, particularly those surrounding SRH, is critical to fostering a society where individuals can make informed decisions and lead healthy lives. These taboos, deeply rooted in cultural, religious, and societal norms, pose significant challenges to individuals and communities, with wide-ranging implications for health, education, and socioeconomic development [39-42]. Addressing these taboos demands comprehensive strategies incorporating education, policy changes, and community engagement [43]. By fostering open dialogue and challenging stigmatizing norms, societies can enhance health outcomes, empower individuals, and promote social and economic development. Efforts to overcome these barriers improve individual well-being and contribute to more excellent stability and prosperity of communities [44-45].

Individual impacts: (a) Reduced access to health services: Taboos surrounding SRH often lead to reduced access to essential health services. In many communities, individuals may feel ashamed or stigmatized when seeking contraception, STI testing, or other sexual health services. Research has shown that stigma and embarrassment deter young people from accessing sexual health clinics, leading to higher rates of untreated STIs and unplanned pregnancies [46-48].

(b) Mental health consequences: Stigmatization and societal shaming of SRH issues can result in profound mental health consequences. Individuals who face discrimination or internalize negative societal attitudes often experience anxiety, depression, and lowered self-esteem. For instance, people diagnosed with HIV/AIDS frequently confront severe stigma, which exacerbates psychological distress and can impede treatment adherence [49].

(c) Inadequate menstrual hygiene management: Cultural taboos surrounding menstruation often lead to inadequate menstrual hygiene management, as observed in numerous developing countries. In these regions, girls frequently miss school because of a lack of proper facilities, sanitary products, and supportive environments for managing their menstruation. This absence of adequate menstrual hygiene facilities and education is intricately linked to broader water, sanitation, and hygiene (WASH) issues. Without access to clean water and sanitation, maintaining menstrual hygiene becomes exceedingly tricky, further stigmatizing menstruation and marginalizing young girls [50-52]. Consequently, the educational opportunities for these girls are severely impacted, leading to higher dropout rates. This not only diminishes their academic prospects but also perpetuates cycles of poverty and entrenches gender inequality. By addressing menstrual hygiene and WASH issues comprehensively, we can empower girls to stay in school, fostering a more inclusive and equitable society where both men and women can contribute fully [53-55].

(d) Sexual and reproductive autonomy: The taboo regarding SRH can severely undermine individual autonomy. Women may struggle to make informed choices about their reproductive health due to societal pressure and misinformation. Deshpande et al. (2018) reported that around “60% of girls used sanitary pads, and the rest used cloth pieces” [56]. Cultural norms that valorize large families and discourage contraception can lead to unwanted pregnancies and unsafe abortions, further endangering women’s health [57].

Community impacts: (a) Public health burdens: Taboos contribute significantly to public health burdens by inhibiting open discussion and education on sexual health topics. This lack of awareness and knowledge fosters environments where STIs, including HIV, can spread rapidly. Communities that stigmatize condom use or discourage open dialogue on sexual health practices see higher rates of these infections [35].

(b) Education and empowerment: The persistence of taboos can impede comprehensive sex education, leaving young people ill-equipped to make safe and informed decisions regarding their sexual health. Educational programs that embrace open, inclusive, and factual discussions about sex and reproductive health are crucial to empowering youth and reducing health disparities. Countries with comprehensive sex education report lower rates of teenage pregnancies and STIs, underscoring the importance of overcoming taboos for educational success [57-61].

(c) Economic development: Taboos around SRH can hinder economic development by limiting women’s participation in the workforce. Health complications from repeated pregnancies, unsafe abortions, and untreated reproductive health issues significantly reduce women’s productivity and economic contributions. Studies indicate that improving women’s health correlates with better financial outcomes for families and communities [62].

(d) Social equity and human rights: Taboos around SRH often exacerbate social inequities and human rights violations. Marginalized groups, including women, LGBTQ+ individuals, and those living with disabilities, face compounded stigma and barriers to accessing necessary health services. Promoting equity requires dismantling these taboos and creating inclusive spaces that respect and uphold the health rights of all individuals [63]. SRH taboos disproportionately affect women and girls, limiting their autonomy and access to necessary health services. Cultural norms that prioritize male authority over female reproductive choices can restrict women’s ability to seek contraceptive services or safe abortion care. A study by Rahman (2012) found that women in patriarchal societies are less likely to use contraceptives and more likely to experience unplanned pregnancies, contributing to higher maternal morbidity and mortality rates [64].

The Landscape of Taboos in Sexual and Reproductive Health

Prevalent stigmatization and secrecy surrounding sexual and reproductive health topics: SRH topics are often shrouded in secrecy and stigmatization, driven by deeply entrenched cultural, religious, and societal norms. This pervasive silence has significant implications for individuals and communities, impacting health outcomes, access to care, and overall well-being. Cultural and religious beliefs play a crucial role in shaping attitudes toward SRH. Many cultures regard topics such as menstruation, contraception, and sexual orientation as taboo because they challenge traditional norms of purity, morality, and gender roles. For example, in conservative religious communities, premarital sex and contraception may be viewed as morally objectionable, leading to their stigmatization [65,66]. In societies where traditional beliefs and practices dominate, discussions about SRH can be seen as inappropriate or shameful, perpetuating secrecy and misinformation [67]. The stigmatization of SRH topics often reflects broader gender and power dynamics within society. Discussions about SRH frequently disproportionately affect women and marginalized groups. Women’s sexuality has historically been controlled and regulated by patriarchal structures, with societal norms often discouraging open discussion and self-advocacy [38,42].

Consequences of stigma and secrecy: (a) Health implications: The stigmatization and secrecy surrounding SRH topics have direct and harmful effects on health outcomes. Fear of judgment and discrimination deters individuals from seeking essential health services [68]. For instance, young people and unmarried women often avoid clinics due to the stigma associated with premarital sex, resulting in untreated STIs and unplanned pregnancies [6,69]. Similarly, the stigma surrounding abortion leads many women to seek unsafe procedures, contributing to maternal morbidity and mortality [2].

(b) Mental health impact: Stigma and secrecy also take a toll on mental health. Individuals grappling with stigmatized SRH issues, such as infertility, STIs, or non-heteronormative sexual orientations, often experience anxiety, depression, and low self-esteem. The lack of open discussion and support can exacerbate feelings of isolation and helplessness [70].

(c) Delayed or foregone medical treatment: Stigma and secrecy around SRH can delay seeking medical care. Individuals with symptoms of STIs, including HIV, may avoid seeking diagnosis and treatment due to fear of social stigma [47,71]. This delay can lead to more advanced disease stages, which are more challenging to treat and have more severe health consequences. For example, untreated STIs can lead to complications such as infertility, chronic pain, and an increased risk of HIV transmission [72,73].

(d) Educational barriers: The reluctance to address SRH topics openly hampers education efforts. In many regions, sex education is limited or absent due to cultural or religious opposition. This lack of information leaves young people unprepared to make informed decisions about their sexual health, increasing their vulnerability to STIs, unintended pregnancies, and sexual violence [13].

(e) Socioeconomic effects: The economic implications of SRH stigma are significant. Poor sexual health can limit educational and employment opportunities, particularly for women. For instance, girls who miss school due to inadequate menstrual hygiene management are less likely to complete their education, adversely affecting their future income and economic stability [11,12].

The Compounded Effects of Multiple Layers of Disadvantages in Sexual and Reproductive Health

The intersectionality framework: The concept of intersecting or compounded disadvantages arises from recognizing that various forms of inequality do not operate in isolation. Instead, they interact, creating amplified challenges for individuals affected by multiple layers of marginality. In the realm of SRH, compounded disadvantages significantly affect access to care, health outcomes, and overall well-being [74]. Intersectionality is a framework for understanding how various forms of social stratification, such as race, gender, class, and sexuality, intersect to create different modes of discrimination and privilege [75]. This perspective is essential for analyzing the compounded effects of multiple disadvantages in SRH as it acknowledges the complex, intertwined nature of social inequalities. This discussion explores how overlapping layers of disadvantage can exacerbate health disparities, mainly focusing on the interplay of gender, socioeconomic status, racial or ethnic background, sexual orientation, and geographic location.

Gender and socioeconomic status: Gender and socioeconomic status are two primary factors influencing SRH outcomes [76]. Women from lower socioeconomic backgrounds often face more substantial barriers to accessing SRH services due to financial constraints, lack of healthcare facilities, and lower levels of education. For example, the high cost of contraceptives can deter usage among economically disadvantaged women, leading to higher rates of unintended pregnancies and maternal mortality [77,78]. For example, in many low-income countries, adolescent girls from poorer households are less likely to attend school and receive comprehensive sex education. This lack of education correlates with higher rates of early marriage, teenage pregnancy, and adverse reproductive health outcomes. The United Nations Population Fund (UNFPA) reports that girls in the poorest quintile are twice as likely to give birth before 18 compared to those in the wealthiest quintile [79].

Race, ethnicity, and gender: Racial and ethnic minorities often experience heightened SRH disparities due to systemic racism and discrimination. When combined with gender, these disadvantages become even more pronounced. Women of color in many societies face discriminatory healthcare practices, limited access to culturally competent care, and socioeconomic barriers that impede their reproductive health [80]. For example, in the United States, Black women are more likely to experience maternal mortality compared to their white counterparts, with rates three to four times higher [81]. Factors such as implicit bias in healthcare, unequal access to prenatal care, and higher poverty rates contribute to this disparity. The compounded disadvantage of being both a racial minority and a woman creates significant barriers to achieving optimal reproductive health.

Geographic location: A critical role in SRH outcomes depends on geographic location, with rural areas often lacking essential health services. Women and minorities living in rural or remote locations face compounded barriers, including extended distances to healthcare facilities, shortage of trained providers, and limited availability of reproductive health supplies [82]. For example, women from lower castes or tribal communities are less likely to have access to prenatal care, skilled birth attendants, and contraceptive services in rural India. The compounded effects of caste-based discrimination, rural isolation, and economic disadvantage contribute to higher rates of maternal and infant mortality in these populations [83].

Case Studies and Examples From Diverse Cultural and Regional Contexts in Sexual and Reproductive Health

Exploring case studies from diverse cultural and regional contexts provides a deeper understanding of how taboos in SRH manifest and are addressed. These examples highlight the unique challenges different communities face and the innovative strategies employed to overcome stigmatization and improve health outcomes.

Case study 1 regarding menstruation taboos in Nepal: In rural Nepal, menstruation is surrounded by severe taboos. The practice of “Chhaupadi,” where menstruating women are considered impure and are isolated in small huts or sheds, is both culturally and religiously rooted. This period of isolation often leads to significant health risks, including exposure to extreme weather conditions, animal attacks, and a lack of proper sanitation, which can cause infections and even death [84]. Local NGOs and international organizations have launched awareness campaigns and education programs to combat these harmful practices. For example, the NGO Restless Development has implemented community dialogues and training sessions that include both men and women to change perceptions around menstruation [85]. These efforts have started to shift attitudes, with some communities abandoning Chhaupadi and adopting more hygienic menstrual management practices.

Case study 2 concerning comprehensive sex education in the Netherlands: It is often cited as a model for comprehensive sex education indicted comprehensively in the Netherlands. Dutch schools provide age-appropriate, holistic sex education from primary school through secondary school. The curriculum includes information on anatomy, reproduction, contraception, consent, and sexual diversity [86]. This open and inclusive approach has contributed to the Netherlands having some of the lowest rates of teenage pregnancies and STIs in the world [87]. A vital component of the Dutch approach is emphasizing open communication and normalization of sexual health topics. Parents, educators, and healthcare providers are encouraged to talk openly about these issues, helping to destigmatize them. This case study illustrates how providing accurate information and fostering an environment of openness can lead to positive health outcomes.

Case study 3 concerning stigma and HIV/AIDS in South Africa: One of the highest HIV prevalence rates globally was observed in South Africa. The stigma associated with HIV/AIDS remains a significant barrier to testing, treatment, and prevention. In many communities, HIV is linked to promiscuity and moral failure, leading to discrimination and social ostracization [88]. To address this, various initiatives have been launched to reduce stigma and increase access to care. For instance, the “LoveLife” campaign combines media outreach with community-based programs focusing on youth empowerment and education. Using relatable media content and peer education models, LoveLife encourages young people to get tested and seek treatment without fear of stigma. This approach has been effective in increasing awareness and reducing some of the misconceptions about HIV/AIDS [89].

Case study 4 regarding menstrual hygiene in Uganda: Menstrual hygiene is an outstanding challenge due to cultural taboos, limited access to sanitary products, and inadequate facilities in Uganda. Many girls miss school during menstruation, which impacts their educational attainment and future opportunities [90]. The NGO WoMena addresses these issues by promoting menstrual cups and reusable pads as sustainable menstrual hygiene solutions. They conduct educational workshops in schools and communities to break the silence around menstruation and teach proper hygiene practices. This initiative has provided girls with reliable menstrual products and empowered them to stay in school and advocate for their rights [91].

Case study 5 regarding sexual education and reproductive health in Malaysia: Sociocultural and religious conservatism poses significant challenges to SRH in Malaysia. The predominance of Islam and traditional values contributes to taboos around SRH discussions, leading to limited sex education and high rates of teenage pregnancies and STIs [92,93]. Efforts by organizations such as the Federation of Reproductive Health Associations, Malaysia (FRHAM) and the Reproductive Rights Advocacy Alliance Malaysia (RRAAM) have been pivotal. Initiatives include comprehensive sex education pilot programs in schools, youth-friendly clinics, and digital platforms like “SPOT” that offer confidential SRH information [94]. Community dialogues also engage parents and religious leaders to foster supportive environments. Despite successes, challenges remain in scaling these programs due to resistance and funding constraints. Future efforts must focus on broader implementation and increased advocacy for comprehensive SRH education and services.

Case study 6 on family planning in the Philippines: Sociocultural and religious influences, especially from the Catholic Church, have historically restricted access to family planning services in the Philippines. The passage of the Responsible Parenthood and Reproductive Health Act of 2012 aimed to improve access to contraception and reproductive health education, but implementation has faced resistance [95]. NGOs such as the Philippine Legislators’ Committee on Population and Development (PLCPD) and the Likhaan Center for Women’s Health have been instrumental in promoting the law and providing community-based services. Initiatives such as the “Usapan” series offer safe, structured dialogues about family planning in local communities. These efforts have led to increased contraceptive use and a decline in fertility rates, although challenges remain due to funding and ongoing opposition from conservative sectors. Continued advocacy and community engagement are essential for advancing family planning and reproductive health rights in the country.

Gaps and Challenges in Addressing Taboos in Sexual and Reproductive Health

**Figure 3:**
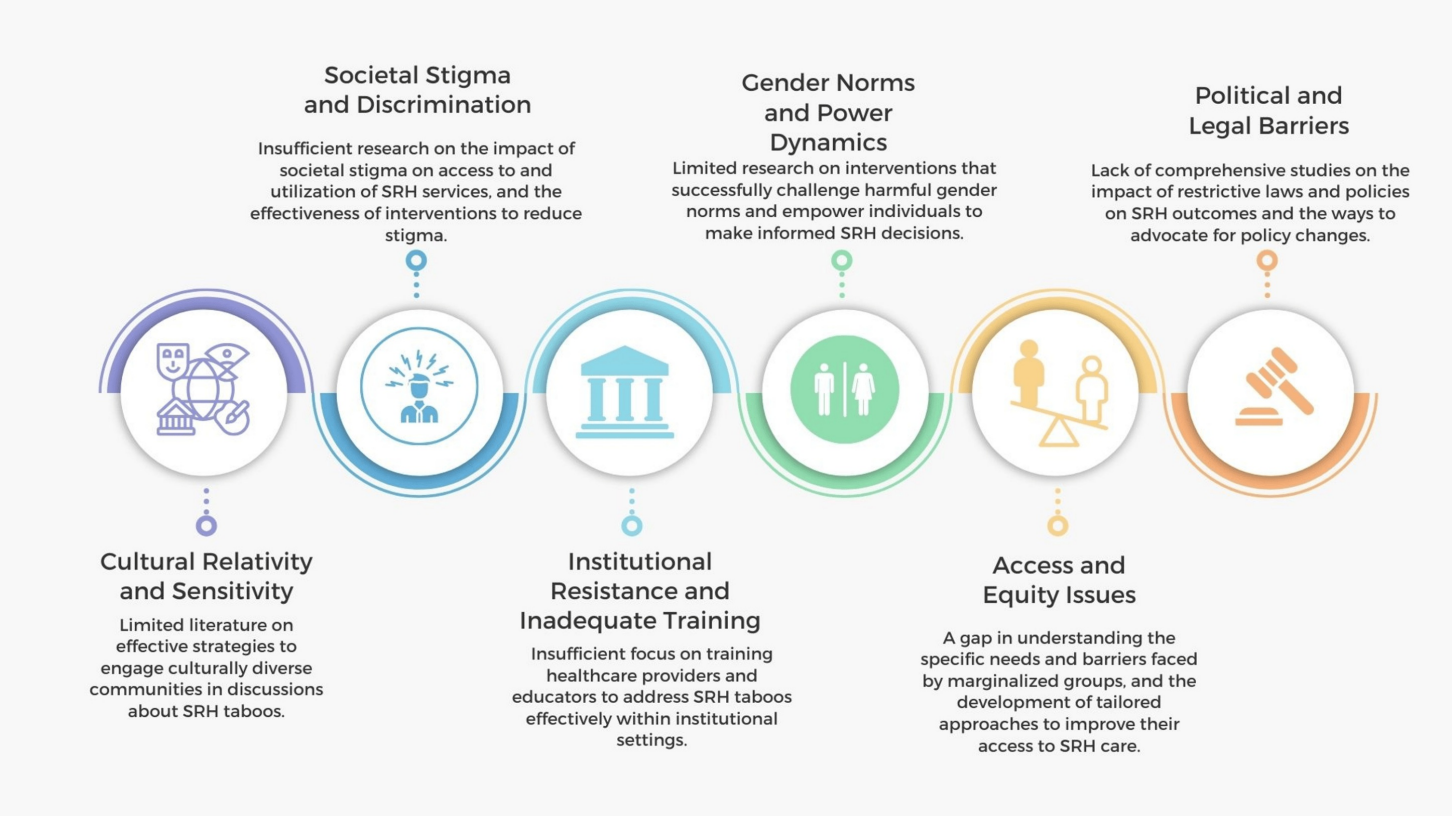
Gaps and challenges in addressing taboos in sexual and reproductive health. Image credit: Nor Faiza Mohd. Tohit.

Addressing taboos in SRH is vital for improving public health outcomes, yet numerous gaps and challenges persist (Figure 3). These obstacles arise from cultural, societal, political, and institutional factors that collectively hinder the progress toward comprehensive and practical SRH education, services, and policies. Addressing these gaps and challenges is critical for creating an environment where SRH can be discussed openly and comprehensively, leading to better health outcomes and reduced stigma.

Strategies for Change

Addressing long-standing challenges and gaps in SRH requires comprehensive policy changes, improved healthcare practices, and enhanced public discourse. These efforts must emphasize inclusivity, human rights, and comprehensive healthcare to overcome the barriers posed by cultural, religious, and societal taboos.

Policy recommendations: (a) Comprehensive sex education: One of the most critical policy changes is the mandatory implementation of comprehensive sex education (CSE) in schools. This curriculum should be age-appropriate, medically accurate, and inclusive, covering essential topics such as contraception, consent, healthy relationships, sexual orientation, and gender identity. Integrating best practices from countries like the Netherlands, where CSE is a standard part of the education system, can provide a robust framework. Studies have shown that such programs lead to improved SRH outcomes, including lower rates of unintended pregnancies and STIs [96,97]. Policies should support professional development opportunities for educators to ensure they are well-prepared to deliver this comprehensive curriculum effectively [98].

(b) Access to SRH services: Policies must prioritize integrating SRH services into primary healthcare to ensure they are accessible, affordable, and stigma-free. This includes subsidized SRH services for low-income populations to bridge the economic gap that often limits access to essential care [99]. Governments should legislate to make contraception, STI testing, and counseling services widely available and affordable and integrate these services into community health centers and mobile clinics, especially in rural and underserved areas [100,101]. Offering mobile and community-based clinics in underserved areas can bridge the gap for rural populations and those with limited transportation options. Additionally, telemedicine should be expanded to provide confidential and accessible SRH consultations and services [92,102,103].

(c) Anti-discrimination frameworks: Robust anti-discrimination laws need to be enacted and enforced to protect marginalized groups such as women and racial minorities [104]. These laws should explicitly prohibit discrimination in accessing healthcare services and education, ensuring equitable treatment. Policy frameworks should include mandatory sensitivity and cultural competence training for healthcare providers to mitigate biases and provide respectful and effective care for diverse populations [105]. Furthermore, there should be mechanisms for tracking and addressing instances of discrimination in healthcare settings to ensure accountability and continuous improvement [106].

**Figure 4:**
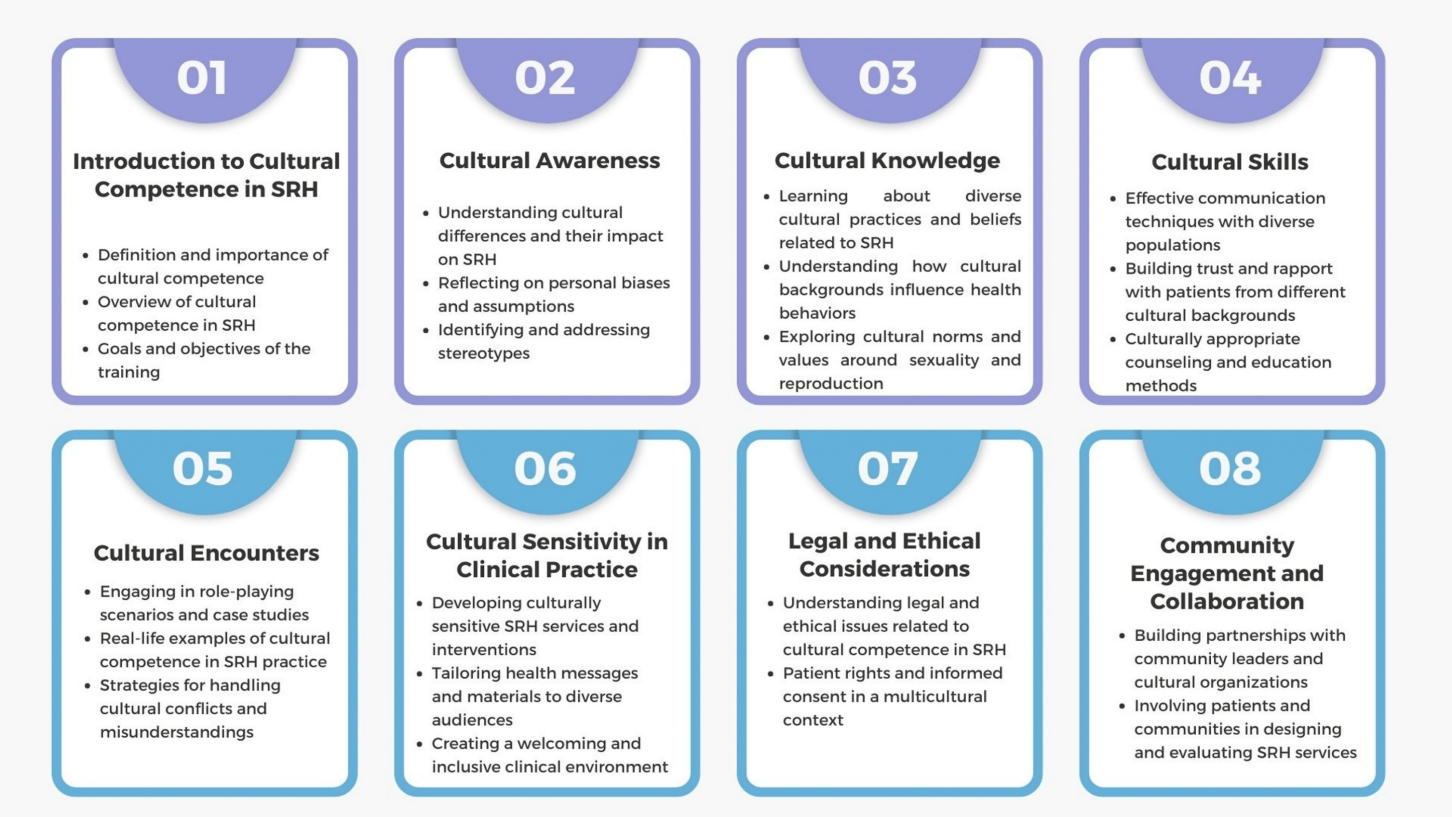
Suggested modules for cultural competence training. Image credit: Nor Faiza Mohd. Tohit.

Healthcare practice recommendations: (a) Cultural competence training: Cultural competence training is essential for healthcare providers to deliver practical, respectful, and empathetic care to diverse patient populations. This training involves educating providers about different cultural beliefs, practices, and values related to health and illness, ensuring they understand and respect the unique needs of each patient [107,108]. Cultural competence encompasses awareness of the sociocultural factors influencing health behaviors and outcomes, such as language barriers, religious beliefs, and socioeconomic status. Training programs typically include modules on communication skills, legal and ethical considerations, and culturally appropriate care practices (Figure 4). Providers learn to avoid stereotypes and assumptions, instead approaching each patient as an individual with unique needs and preferences [109]. Sensitivity to cultural differences in areas such as SRH can significantly improve provider-patient interactions and health outcomes. For example, understanding cultural taboos around discussing sexual health can help providers create a more comfortable environment for patients to share their concerns and access necessary services [41,110]. The benefits of cultural competence training extend to improving patient trust and satisfaction, reducing health disparities, and enhancing the overall quality of care. Health organizations should implement continuous cultural competence training to keep healthcare providers updated on the evolving demographic and cultural landscape [111]. By fostering a culturally inclusive health system, we can promote equitable access to care and better health outcomes for all individuals, regardless of their cultural background [112,113].

**Figure 5:**
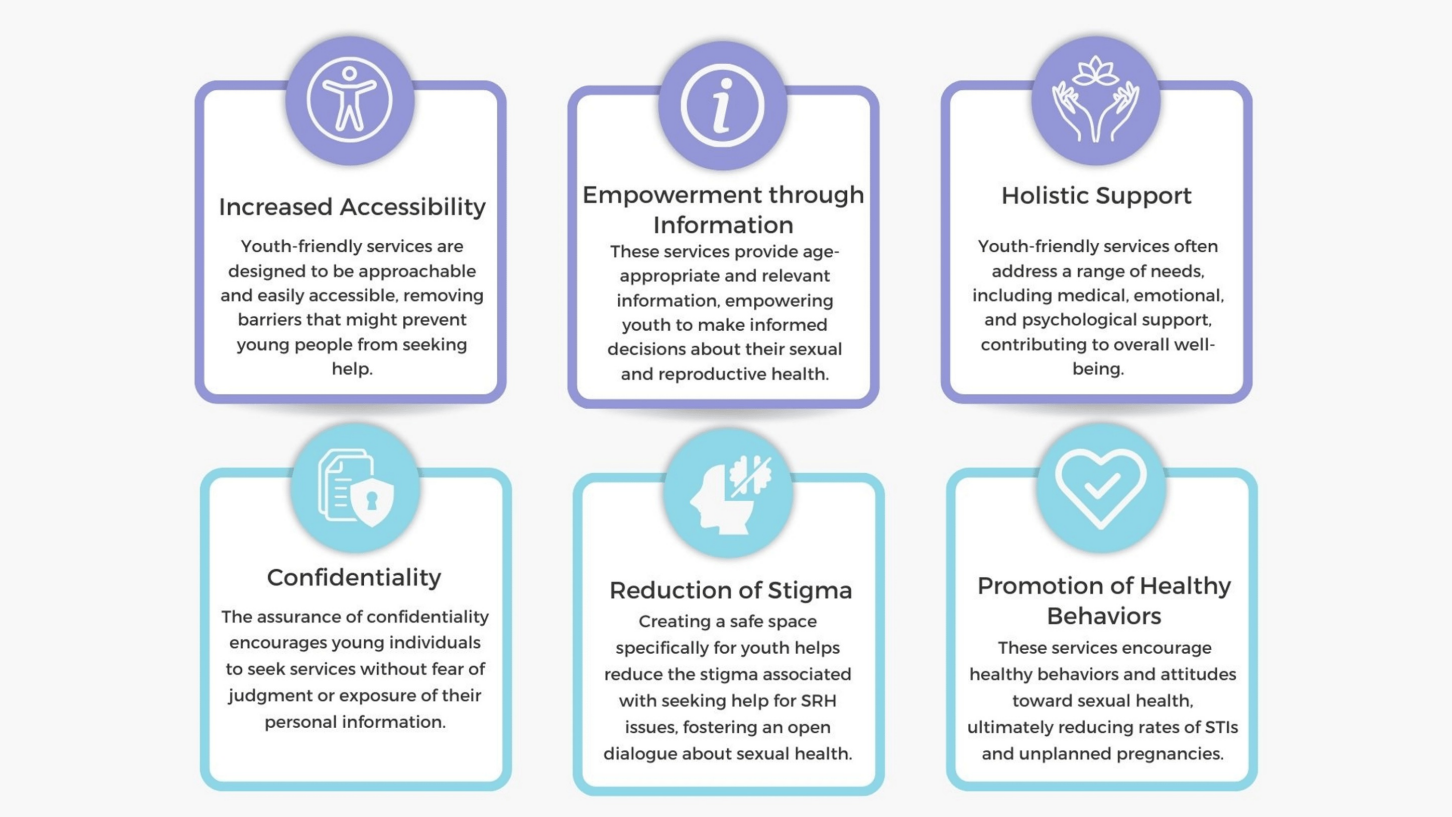
Advantages of providing youth-friendly and confidential services in sexual and reproductive health. Image credit: Nor Faiza Mohd. Tohit.

**Figure 6:**
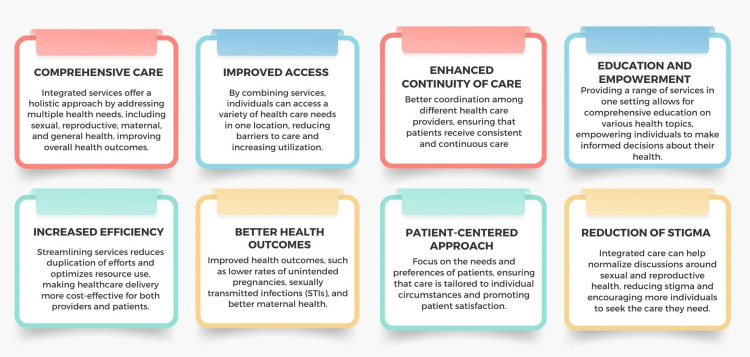
Advantages of providing integrated services in sexual and reproductive health. Image credit: Nor Faiza Mohd. Tohit.

(b) Youth-friendly and confidential services: Creating policies supporting youth-friendly SRH services can significantly improve healthcare access for adolescents and young adults (Figure 5). These services must be confidential, easily accessible, and tailored to meet the specific needs of young people [114-116]. Governments should encourage the deployment of adolescent health services in schools and community centers and provide training for healthcare professionals in adolescent health to ensure an empathetic and supportive environment for youth seeking care [117]. Establishing youth-friendly SRH services can encourage young people to seek information and care without fear of stigma. Youth-friendly and confidential services ensure that young people feel welcomed, safe, and respected when seeking support. It is essential to recognize that young people often face unique challenges and barriers when accessing services, whether related to health, education, or social support [118]. One key aspect of making services youth-friendly involves creating a physically and emotionally safe environment. This means having spaces that are private, comfortable, and non-judgmental [108,109]. Additionally, staff should be trained to interact with young people in a supportive and understanding manner, recognizing the diverse backgrounds and experiences they bring [95]. Confidentiality is another fundamental component. Young people must trust that their personal information will be kept private and only shared with their consent unless severe concerns about their safety or well-being exist. Clear communication about confidentiality policies can help to build this trust [119]. It is also vital to provide services tailored to the specific needs of young people. This might include flexible hours to accommodate school schedules, culturally appropriate resources, and technology integration for easier access to information and support. Engaging young people in designing and evaluating services can further enhance their effectiveness [120]. Gathering feedback through surveys, focus groups, or youth advisory boards can provide valuable insights into how services can be improved to better meet the needs of this demographic. Anticipating and addressing potential barriers is essential. For instance, providing transportation or leveraging mobile clinics can help overcome issues related to accessibility [47]. Additionally, offering services at low or no cost can alleviate financial constraints that may prevent young people from seeking help. Finally, it is essential to evaluate and improve services continuously. Regular staff training, updated resources, and ongoing dialogue with young clients ensure that the services remain relevant, effective, and responsive to the evolving needs of youth [121-124]. By focusing on these elements, we can create an environment where young people feel empowered and supported to seek the help they need, ultimately leading to better outcomes in health, education, and overall well-being [45].

(c) Integrated care services: SRH requires integrated care services, which combine multiple facets of health into a cohesive system, comprehensively addressing physical, mental, and social well-being [125]. This model ensures that SRH services, such as family planning, STI prevention, maternal health, and mental health, are coordinated. Co-locating services within a single facility simplifies access, reducing logistical barriers and improving patient convenience [126,127]. Integrating mental health services with SRH care addresses the psychological impacts of stigma and discrimination associated with SRH issues. Holistic care models emphasizing prevention and treatment can improve overall health outcomes [128]. A patient-centered approach is central to integrated care, creating tailored care plans that recognize the interplay between different health aspects. For instance, integrated mental health counseling in family planning services can address psychological barriers to contraceptive use. Such comprehensive care models have been shown to enhance health outcomes; a notable example is reduced mother-to-child HIV transmission rates through the integration of maternal health and HIV services [129]. Interprofessional collaboration among various healthcare providers ensures that care is continuous and coordinated, addressing social determinants of health such as socioeconomic status and education. Effective integrated care will offer multiple advantages (Figure 6). Nevertheless, it requires supportive policies, adequate infrastructure, and training for healthcare providers.

Public discourse recommendations: (a) Public health campaigns: Investing in public health campaigns that normalize open discussions about SRH and challenge existing taboos is essential [130,131]. These campaigns should use mainstream media, social media, and community outreach to disseminate accurate information and promote positive attitudes toward SRH. Public figures, influencers, and respected community leaders can be instrumental in these efforts, helping to break down stigmas and encourage community acceptance. Campaigns featuring public figures and influencers can amplify these messages and promote positive attitudes toward SRH [132]. Governments should allocate dedicated funding for these campaigns to ensure they are widespread and sustained.

**Figure 7:**
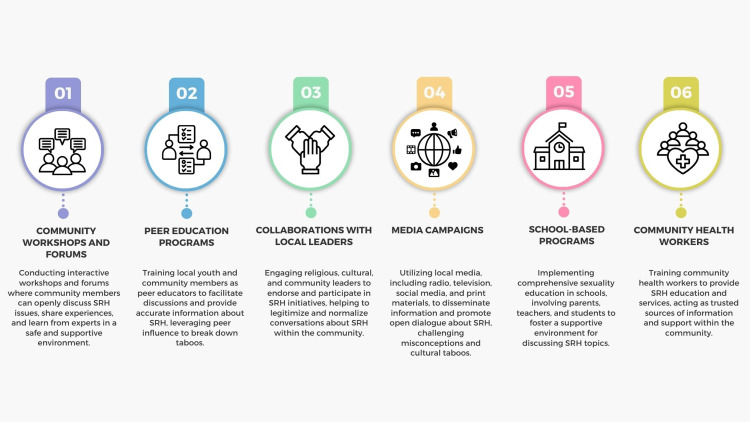
Methods of performing community engagement to address taboos around sexual and reproductive health. Image credit: Nor Faiza Mohd. Tohit.

(b) Community engagement: Various methods can be used to conduct community-based initiatives that involve parents, religious leaders, and other stakeholders (Figure 7). Engaging these groups in dialogue can help tailor culturally appropriate and acceptable programs, facilitating community buy-in and support [133].

Community engagement in SRH is crucial for creating health programs that are culturally sensitive, effective, and widely accepted. It actively involves community members in the planning, implementing, and evaluating SRH initiatives. Engaging with key stakeholders such as religious leaders, parents, youth, and local organizations helps tailor interventions to meet the specific needs and values of the community [134]. By fostering open dialogue and collaboration, community engagement helps reduce the stigma associated with SRH and promotes a supportive environment for discussing these issues [86,135]. For instance, involving religious leaders can help reconcile traditional beliefs with modern SRH practices, improving acceptance and adherence. Engaging youth directly ensures that programs address their unique needs and concerns, making SRH services more relevant and accessible. Furthermore, community-driven approaches enhance trust and ownership, leading to sustained behavioral change and better health outcomes [136]. Programs developed with community input are more likely to be accepted and utilized, leading to increased uptake of services such as contraception and HIV testing. Effective community engagement requires continuous feedback loops, transparency, and mutual respect. Health organizations and policymakers should prioritize community involvement as a cornerstone of designing and implementing SRH programs, ensuring they are inclusive, equitable, and effective [137].

**Figure 8:**
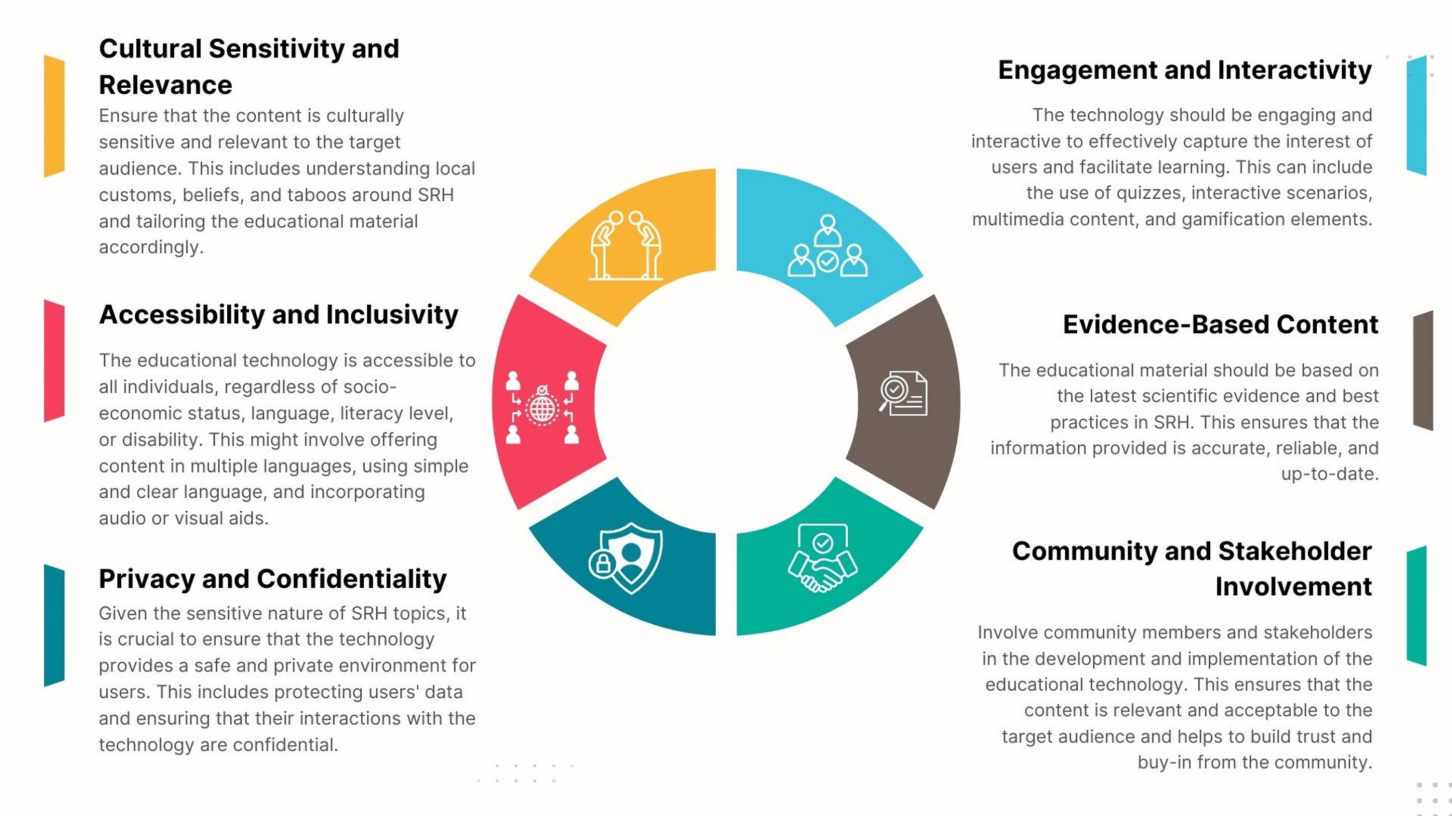
Factors to be considered when implementing educational technology to address taboos around sexual and reproductive health. Image credit: Nor Faiza Mohd. Tohit.

(c) Educational technology: Utilizing technology and digital platforms can significantly overcome traditional barriers to SRH education by providing accessible, confidential, and interactive learning opportunities. Face-to-face SRH education may be hindered in many environments by social stigma, limited resources, or a lack of qualified educators [138]. These constraints can leave many individuals, particularly youth, without the essential knowledge to make informed decisions about their health and well-being [139]. One of the primary advantages of employing digital platforms is the potential to reach a much broader audience than traditional methods. Online resources, mobile apps, and social media campaigns can disseminate accurate information widely, reaching diverse audiences, particularly youth, who may have limited access to SRH education in traditional settings [140]. For example, a teenager in a remote area or a conservative community where sexual health topics are taboo can access vital information through their smartphone or computer, ensuring they are not left out of the educational loop. These digital tools can also provide interactive and personalized guidance tailored to individual needs [140,141]. For instance, online platforms can host anonymous Q&A sessions where users can ask sensitive questions without fear of judgment or exposure. Mobile apps can offer tailored recommendations based on personal health data, age, or cultural background, making the learning experience more relevant and engaging. This anonymity and personalization are particularly empowering, as they encourage individuals to seek and absorb information they might otherwise shy away from. Another crucial aspect of digital SRH education is the ability to deliver culturally relevant content. Educational tools can be adapted to reflect various regions’ cultural and societal norms, making the information more relatable and easier to understand. This is vital in creating inclusive educational environments that respect and acknowledge diversity, ultimately fostering a more accepting and knowledgeable society. Empowering individuals to make informed decisions about their health is the cornerstone of effective SRH education [19,112]. Access to reliable, clear, and comprehensive information makes people better understand their bodies, recognize health issues, and seek appropriate care when necessary. Technology amplifies this empowerment by breaking down barriers and providing diverse resources that can be accessed anytime and anywhere. Several factors must be considered when implementing educational technology to address SRH taboos (Figure 8). By leveraging the strengths of technology, SRH education can become more inclusive, engaging, and effective in addressing the needs of modern populations. Future initiatives could further enhance this by incorporating newer technologies, such as virtual reality for immersive learning experiences or artificial intelligence, to provide even more personalized guidance [113,114]. The continuous evolution of digital tools holds great promise for the future of SRH education, ensuring that it remains relevant and accessible to all.

Emphasizing Inclusivity, Human Rights, and Comprehensive Healthcare

**Figure 9:**
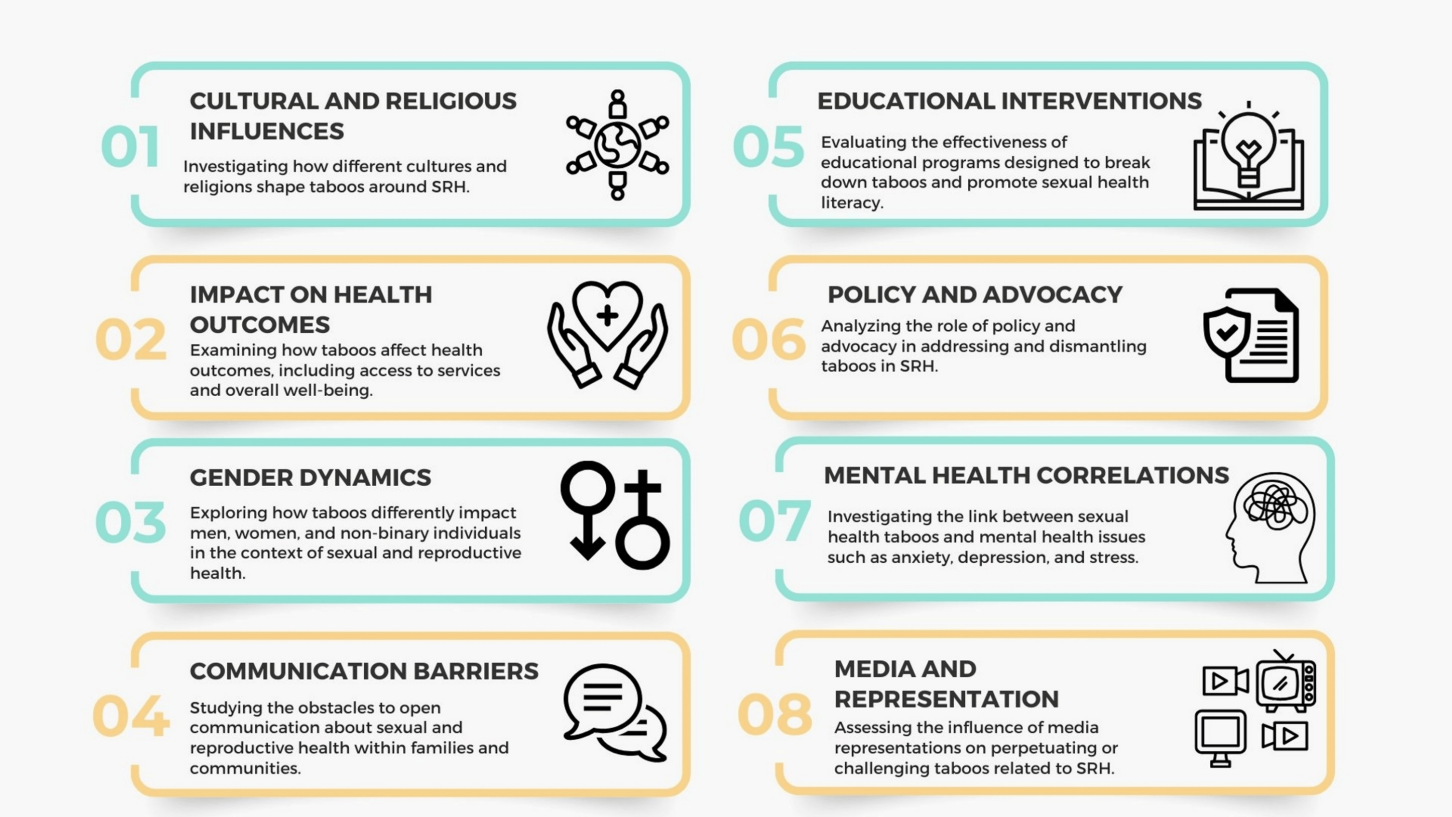
Potential research areas to address the issue of taboos around sexual and reproductive health. Image credit: Nor Faiza Mohd. Tohit.

Inclusivity ensures all individuals, regardless of gender, socioeconomic status, race, sexual orientation, or geographic location, have access to accurate information and quality SRH services [142,143]. Human rights principles should underpin all policies and practices, affirming individuals’ rights to make informed choices about their bodies and health [144-146]. Comprehensive healthcare integrates SRH services into broader health systems, ensuring a continuum of care that addresses physical, mental, and social health aspects. Further research should be carried out to address the gaps and challenges in SRH (Figure 9).

Limitations

**Figure 10:**
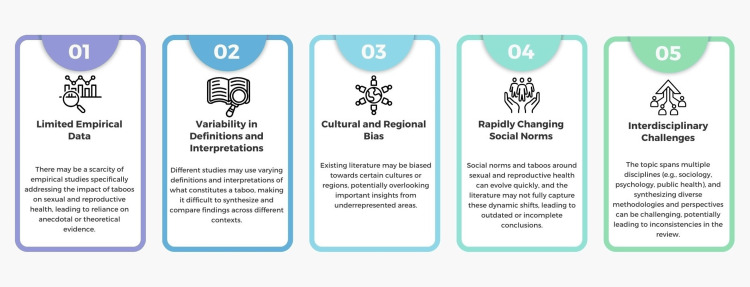
Limitations of the scoping review. Image credit: Nor Faiza Mohd. Tohit.

This scoping review has several limitations (Figure 10). Nevertheless, the information gathered has outlined a crucial point: addressing SRH taboos and barriers requires cohesive efforts across policy-making, healthcare practices, and public engagement. By implementing comprehensive sex education, improving access to inclusive and culturally competent healthcare, and promoting open public discourse, societies can break down the barriers created by taboos, improve health outcomes, and reduce disparities [147,148]. These recommendations, rooted in inclusivity and human rights, provide a framework for creating an environment where SRH is openly discussed, accurately understood, and effectively managed, ultimately enhancing the quality of life for all individuals.

## Conclusions

Addressing and dismantling the taboos surrounding SRH is crucial for advancing public health and ensuring the well-being of all individuals. This comprehensive exploration reveals the extent to which these taboos infiltrate societal attitudes, healthcare practices, and educational systems. The findings of this review highlight the urgent need for a shift toward open, inclusive, and evidence-based conversations and practices in SRH.

Future efforts should focus on the continued implementation of inclusive policies, comprehensive education programs, and fostering a healthcare environment that prioritizes empathy, respect, and open dialogue. Addressing these taboos head-on can pave the way for improved health outcomes, reduced disparities, and enhanced individual well-being. Ultimately, this review serves as a call to action. It urges policymakers, educators, healthcare providers, and communities to engage in meaningful conversations, dismantle harmful taboos, and support the realization of SRH rights for all individuals, regardless of their background or circumstances.
